# Orthodontic treatment needs in the western region of Saudi Arabia: a research report

**DOI:** 10.1186/1746-160X-2-2

**Published:** 2006-01-18

**Authors:** Ali H Hassan

**Affiliations:** 1 P.O. Box 80209, Jeddah 21589, Preventive Dental Sciences Department, Faculty of Dentistry, King Abdulaziz University, Jeddah, Saudi Arabia

## Abstract

**Background:**

Evaluation of self perceived and actual need for orthodontic treatment helps in planning orthodontic services and estimating the required resources and man power. In the present study, the perceptive need as evaluated by patients and the actual need to orthodontic treatment, as assessed by orthodontists, were evaluated at two types of dental practices in the city of Jeddah using the Index of Orthodontic Treatment Need (IOTN).

**Methods:**

A consecutive sample of 743 adults seeking orthodontic treatment at two different types of dental practices in Jeddah; King Abdulaziz University, Faculty of Dentistry (KAAU) (Free treatment) and two private dental polyclinics (PDP) (Paid treatment), was examined for orthodontic treatment need using the dental health component (DHC) of the IOTN. The self-perceived need for orthodontic treatment was also determined using the aesthetic component (AC) of the IOTN. The IOTN score and the incidence of each variable were calculated statistically. AC and DHC categories were compared using the Chi-Square and a correlation between them was assessed using Spearman's correlation test. AC and DHC were also compared between the two types of dental practices using the Chi-Square.

**Results:**

The results revealed that among the 743 patients studied, 60.6% expressed no or slight need for treatment, 23.3% expressed moderate to borderline need and only16.1% thought they needed orthodontic treatment. Comparing these estimates to professional judgments, only 15.2% conformed to little or no need for treatment, 13.2% were assessed as in borderline need and 71.6% were assessed as in need for treatment (p < 0.001). Spearman's correlation test proved no correlation (r = -.045) between the two components. Comparing the AC and the DHC between the KAAU group and PDP group showed significant differences between the two groups (p < 0.001).

**Conclusion:**

Patient's perception to orthodontic treatment does not always correlate with professional assessment. The IOTN is a valid screening tool that should be used in orthodontic clinics for better services especially, in health centers that provide free treatment.

## Background

Orthodontic treatment is an elective treatment that depends on the perception of both the patient and the treating orthodontist. In Saudi Arabia, governmental sectors provide free orthodontic treatment for Saudi citizens. This has generated long waiting lists of patients that can extend for two to four years. Evaluation of self perceived and actual need for orthodontic treatment as well as other factors affecting these needs such as personal, socio-demographic, and psychosocial factors help in planning orthodontic services and estimating the required resources and manpower. Moreover, unnecessary referrals by general practitioners and lengthy waiting lists for orthodontic treatment can be eliminated by limiting free treatment to patients with malocclusions sever enough to warrant treatment [1,2]. It may also predict patients' level of interest and motivation toward the orthodontic treatment, which could help in planning educational programs in schools and media to increase patient's awareness and to overcome obstacles and barriers in seeking treatment [3].

Perceptive or self assessed need to dental care is reported to be associated with certain signs and symptoms [4], socio-demographic factors and satisfaction with previous dental treatment [5,6]. Previous studies have shown differences between patients' and professionals' perception on orthodontic treatment need [8-13]. It seems that normative or actual need as assessed by dental professionals may not be linked to patients' perceptions unless the condition has progressed sufficiently to be symptomatic [7]. Several indices were developed to evaluate malocclusion, such as the IOTN [11], PAR (Peer Assessment Rating Index) [14] and ICON (Index of Complexity, outcome and Need) [15]. The IOTN and the ICON can serve as neutral instruments to determine treatment needs and to allocate financial resources for orthodontic cases [16]. Although the IOTN and the ICON are similar and largely in agreement in measuring treatment needs of patients from different ethnic backgrounds [17], the IOTN has been used extensively in literature to evaluate actual and perceptive treatment needs in different ethnic backgrounds and it seems to be a more popular research tool in the Middle East than the ICON [18-32]. In addition, the IOTN is simpler than the ICON in assessing treatment needs since ICON was designed to measure complexity of treatment in addition to treatment needs [15].

The IOTN is a scoring system for malocclusion, developed by Brook & Shaw (1989) [11]. It consists of two independent components; the DHC, which is a five grade index that records the dental health need for orthodontic treatment, and the AC that records the aesthetic need for orthodontic treatment using a ten grade standardized ranking scale of colored photographs showing different levels of dental attractiveness. In Saudi Arabia, not a single study has been conducted regarding treatment needs among regular orthodontic patients.

The objectives of the present study were:

1- To assess the perceptive and actual treatment needs for orthodontic treatment among subjects seeking orthodontic treatment in the city of Jeddah using the IOTN

2- To compare those subjects attending a governmental dental clinic (KAAU), with those attending PDP utilizing the IOTN.

## Methods

A consecutive sample of 743 subjects (aged 17–24 years) seeking orthodontic treatment was used in this study. The sample was collected from two different types of practices; KAAU (N = 489) and two PDPs in the city of Jeddah (N = 254) during the period of August-November 2004. All subjects were of Arabic descendants and with no history of orthodontic treatment. The treatment at KAAU is free of charge while the treatment at PDPs is quite expensive for the general Saudi population. All subjects who were enrolled in the study signed a consent form. Each subject was examined for orthodontic treatment need using the DHC of the IOTN. Additionally, the self-perceived need for orthodontic treatment was determined by asking each subject to evaluate his or her own attractiveness by comparing it to the standard photographs of the AC of the IOTN. Two examiners were involved in the study, one for the DHC and the other for the AC. The examiners were trained to use the IOTN following the instructions provided with the IOTN materials. The IOTN score and the incidence of each variable were calculated statistically. The sample used in the present study was distribution free and therefore non-parametric tests were used. The AC and DHC categories were compared between the two groups using the Chi-Square. The DHC and the AC were also compared using the Chi-Square and were correlated using Spearman's Correlation coefficient. The protocol of the present study was approved by the Ethical Committee of the Faculty of Dentistry at King Abdulaziz University.

## Results

The highest incidence of orthodontic problems in the current study was for displacement (89.1%), followed by crossbite (44.5%), deep overbite (33.6%), increased overjet (33.6) and openbite (20%). Impaction incidence was relatively low (8.2%). The incidence of cleft lip and palate was 3.9% (Table 1)

**Table 1:** The incidence of the orthodontic problems as assessed by the DHC of the IOTN

**Orthodontic Problem**	**Incidence %**	**Grade 1**	**Grade 2**	**Grade 3**	**Grade 4**	**Grade 5**
Pre/post-normal occlusion	3		3			
Displacement	89.1	3.6	25.5	16.4	43.6	
Crossbite	44.5		14.5	13.6	16.4	
Open bite	20		9.1	6.4	4.5	
Overbite	33.6		18.2	8.2	7.3	
Overjet	33.6		16.4	9.1	5.5	2.7
Reverse overjet	3.6				2.7	0.9
Scissors bite	10.9				10.9	
Partially erupted	9.1				9.1	
Impaction	8.2					8.2
Submerged deciduous	0					0
Supernumerary	.9				0.9	
Hypodontia	3.6				3.6	0
Cleft lip-Palate	3.9					3.9

**Figure 1:**
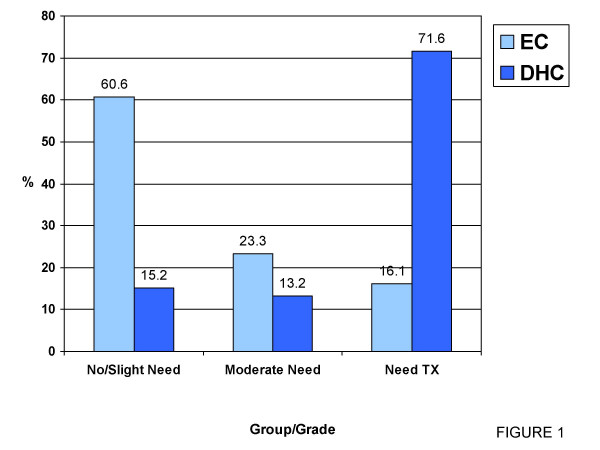
Graphical representation table 2.

Results of the AC revealed that among the 743 patients studied, 60.6% expressed no or slight need for treatment, while 23.3% expressed moderate to borderline need and 16.1% expressed great need for orthodontic treatment. Comparing these estimates to professional judgments using the Chi Square, the DHC was significantly (<0.001) different from the AC in the three groups; 15.2% had little to no treatment need (grades I & II), 13.2% had borderline treatment need (grade III) and 71.6% had a great need for orthodontic treatment (grade IV & V) (Table 2 & Figure 1). Spearman's correlation between the AC and DHC proved no correlation (r = -0.045) between the two components.

**Table 2:** Comparison of the AC and the DHC overall grades

**Group/Grade**	**AC**		**DHC**		**Chi-Square**	**P-value**
			
	*%*	*N*	*%*	*N*		
No/Slight Need	60.6	450	15.2	113	201.007	0.000*
Moderate need	23.3	173	13.2	98	20.756	0.000*
Need TX	16.1	119	71.6	532	262.011	0.000*
Total		742		743		

* Significant at p < 0.001 at 1 d.f.

**Figure 2:**
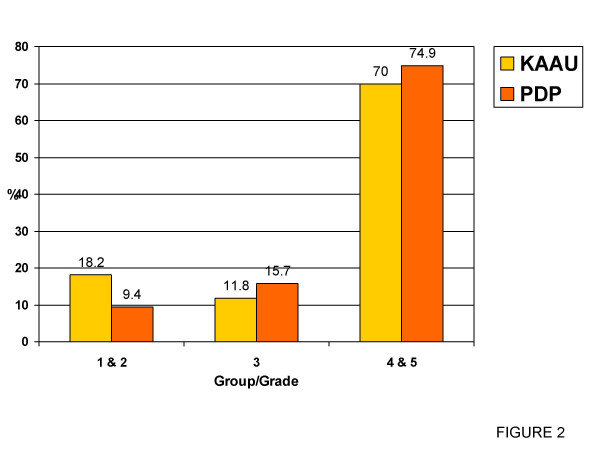
Graphical representation of table 3.

Comparing the grades of DHC between the KAAU group and the PDP group (Table 3 & Figure 2) revealed that the proportion of the sample estimated to have little to no treatment need (Grade I & II) was significantly higher in the KAAU group (18.2%) than that of PDP Group (9.4%) (p < 0.001). The border line proportion (Grade III) was insignificantly different between the two groups. The proportion of the sample estimated to have a great treatment need was significantly higher in the PDP group than the KAAU group (p < 0.001).

**Table 3:** Comparison of the proportions of the two samples estimated to need orthodontic treatment (DHC)

**Group/Grade**	**KAAU**		**PDP**		**Chi-Square**	**P-value**
			
	*%*	*N*	*%*	*N*		
Little/No TX. (Gr.I&II)	18.2	89	9.4	24	37.389	0.000*
Border line (Gr.III)	11.8	58	15.7	40	3.306	0.069
Need TX. (Gr.IV&V)	70	342	74.9	190	34.429	0.000*
Total		489		254		

* Significant at p < 0.001

**Figure 3:**
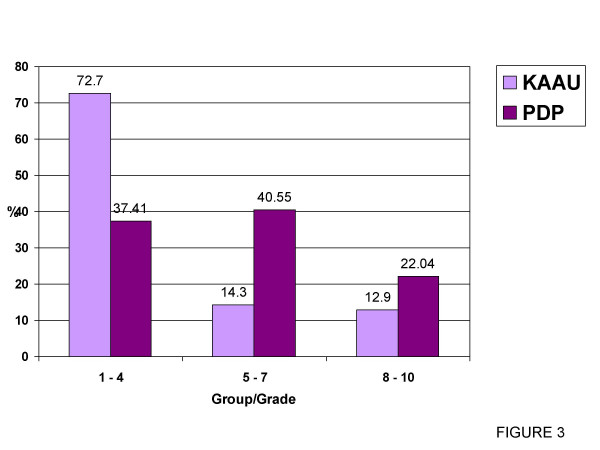
Graphical representation of table 4.

Comparing the grades of the AC between the KAAU group and the PDP group (Table 4 & Figure 3) revealed significant differences between the two groups; no or slight treatment need was higher in the KAAU group (72.7%) than PDP group (37.4%) and border line and great treatment needs were higher in the PDP group (40.55% & 22.04% respectively) than in the KAAU group (14.3% & 12.9% respectively).

**Table 4:** Comparison of the Perceptive Need to Orthodontic Treatment (AC) between the KAAU group and PDP group

**Group/Grade**	**KAAU**		**PDP**		**Chi-Square**	**P-value**
			
	*%*	*N*	*%*	*N*		
No/Slight need (Gr.1–4)	72.7	355	37.41	95	150.222	0.000*
Moderate/Border line (Gr.5–7)	14.3	70	40.55	103	6.295	0.000*
Need treatment (Gr.8–10)	12.9	63	22.04	56	0.412	0.521
Total		488		254		

* Significant at p < 0.001 at 1 d.f.

## Discussion

The results of the DHC shed some light on the pattern of malocclusion that is seen in the city of Jeddah, Saudi Arabia, which is dominant mainly of displacement, crossbite, deep bite and increased overjet. However, larger scale studies are required to evaluate the actual pattern of malocclusion in the western region of Saudi Arabia via conducting survey studies on a random sample. The age group targeted in the present study was different than most of the previous studies [1,8,9,13,19], which were conducted on children and adolescents who are less reliable in their perception than adults, especially when using the IOTN which moderately reflects the subjective perception of dental aesthetics and demand for orthodontic treatment [30].

The significant differences between the AC and DHC and the negative weak correlation between the perceptive and actual need for orthodontic treatment indicates a general lack of awareness among the Saudis about the severity of their existing malocclusion. This can be attributed to their weak oral health knowledge as well as parents' neglect towards malocclusion. This is in agreement with several other studies [8-10]. Moreover, the perception of occlusal traits in the buccal segments is generally underestimated by people when compared to those present in the anterior segment [22,23]. The results can also be attributed to the nature of the IOTN itself. The scores of the DHC may have been exaggerated by the rank of displacement, which would give a high score in otherwise normal occlusion. In addition, the standard photographs of the AC do not show common orthodontic problems such as open bite, which represents a relatively high incidence in the studied sample (20%). This may have misled those subjects with openbite in their perception of their malocclusion. Also, there is no evidence of how the severity of those traits is perceived by people. These shortcomings of the IOTN indicate the need to study the appropriateness of the IOTN or ICON as an index for the Saudi Arabians or even to develop a new index that suits such population.

The results have also shown less awareness and appreciation of the severity of malocclusion among patients seeking treatment in a governmental dental clinic such as KAAU, when compared to those paying for their treatment at private dental polyclinics. This could explain the lack of compliance seen among those patients. Little awareness for the actual need for treatment in the KAAU group could be attributed to the free treatment provided, which attracts anyone to seek treatment regardless of the severity of his or her malocclusion. Therefore, it is recommended to use the DHC of the IOTN as a screening tool to reevaluate the waiting lists of patients seeking orthodontic treatment at governmental clinics. This would identify those patients who could benefit the most from such free services and subsequently reduce the long waiting lists at such centers. In addition, the application of minimum charge for treatment at governmental dental clinics can serve the same purpose.

## Conclusion

There is a definitive need for orthodontic treatment among Saudis living in the city of Jeddah, which is not matched with a similar level of perceptive need by the same population. The grades of AC and DHC were significantly different between those patients seeking free treatment at governmental dental centers and those who pay for their treatment at private dental practices.

## Competing interests

The author(s) declare that they have no competing interests.
